# Script for resilience analysis in energy systems: Python programming code and partial associated data of four cogeneration plants

**DOI:** 10.1016/j.dib.2021.106986

**Published:** 2021-03-23

**Authors:** Fellipe Sartori da Silva, José Alexandre Matelli

**Affiliations:** São Paulo State University, School of Engineering, Department of Chemistry and Energy, Guaratinguetá, São Paulo Brazil

**Keywords:** Resilience, Energy system, Cogeneration, System design

## Abstract

This article presents a script developed to evaluate resilience in energy systems. The files corresponding to the system description, simulation and metrics calculation are included in the dataset, as well as partial raw and processed data from the associated paper [1]. The model was developed focusing on covering all cogeneration and power plants, being the user responsible for describing the system, simulating and processing the data in the files here available. In the present work, the steps for the simulation are presented in detail, which contributes to other researchers that are interested in either adopting resilience as one of the possible system analyses or understanding the processes of metrics calculation of the associated paper.

## Specifications Table

**Table:** 

Subject	Safety, Risk, Reliability and Quality
Specific subject area	Resilience in energy systems
Type of data	1 Python programming code (.py extension). 2 Excel files for description of new systems and calculation of the metrics of the obtained data. 5 Excel files containing associated data.
How data were acquired	Systems descriptions were used in a Python script run in Pycharm 2019.2.6.
Data format	The processed data is given by the “Metrics calculation – example”, which is a partial data of the associated paper. The other files contain raw data.
Parameters for data collection	Data is obtained following a methodology started by [2] and continued and developed in the associated paper [1]. It considers a Monte Carlo-based approach, given as input the configuration of the system, probability of component normal operation, component repair probability, number of simulations and system lifetime.
Description of data collection	Systems were described through their components and connections. These files served as input to the simulation program, which generates output files containing the simulated time, resilient time and downtime of each round of simulation. These data were analyzed by an excel file, which calculates the value of the metrics proposed in the methodology.
Data source location	Institution: São Paulo State University, School of Engineering City/Town/Region: Guaratinguetá, São Paulo Country: Brazil
Data accessibility	Repository name: Mendeley Data Data identification number: DOI: 10.17632/fw4ryysk3v.1 Direct URL to data: https://data.mendeley.com/datasets/fw4ryysk3v/1
Related research article	Fellipe Sartori da Silva, José Alexandre Matelli, Resilience in cogeneration systems: metrics for evaluation and influence of design aspects, Reliability Engineering & System Safety, v. 212 p. 107,444, 2021. https://doi.org/10.1016/j.ress.2021.107444

## Value of the Data

• This paper contributes with a script for resilience analysis in energy systems. Any energy generation system can be proposed and analyzed in the view of resilience. The use of the script presented herein can support a decision making in the systems design phase by providing numerical information about seven metrics explained in [1], each one of them presenting data about system resilience.
• Researchers and system designers that propose new power plant configurations can use the presented script to evaluate resilience of these systems during the conceptual design phase, then being able to choose the most stable and reliable configuration from resilience point of view.
• This script allows the prediction of system behavior under multiple failure scenarios. The use of this method in different systems is important for understanding the resilience in the context of energy systems, thus developing the knowledge of preventive actions for keeping the energy generation under extreme conditions.

## 1 Data Description

The provided dataset contains two folders named “Associated data” and “Model for resilience analysis”. The former consists of raw and processed data from associated paper [1], while the latter encloses the excel files which can be used to describe a new system and process simulated data, and a Python file for simulating the systems according to some input parameters given by the user.

In “Associated data”, it can be found examples of the description of four different cogeneration systems, which are those considered in the analysis within the associated paper, named “S#1_info.xlsx”, “S#2_info.xlsx”, “S#3_info.xlsx” and “S#4_info.xlsx”. These files contain information about the connection, function and repair time of the systems components. The numbers in the files names refer to the identification number of each system. It is also located in this folder another excel file named “Metrics calculation – Example.xlsx”, which contains partial processed data from [1]. These data include the simulation input parameters (number of simulations, system expected lifetime, component unsuccessful repair probability and probability of component normal operation), the simulation output data (simulation time, resilient time and downtime), and the calculation of the seven metrics proposed in that paper for each analyzed system.

The “Model for resilience analysis” folder comprehends files adjusted to receive parameters from new users willing to analyze systems from resilience point of view. The system can be described in “S#()_info.xlsx”, in which the user gives information about the system components (connection, function and repair time), and simulated in “resilience analysis.py”, a python file containing the code that simulates the systems in a stochastic failure environment, as described within the associated paper. The simulation will generate an output data comprised in a “.xls” file, which encloses the simulation time, the resilient time and the downtime of each simulation. This file can be processed in “Metrics calculation.xlsx”.

## 2 Experimental Design, Materials and Methods

The main steps for the systems resilience analysis enabled by this article are: system description, simulation, and data processing. Each step is described in detail in the next subsections.

### 2.1 System description

**Fig. 1:**
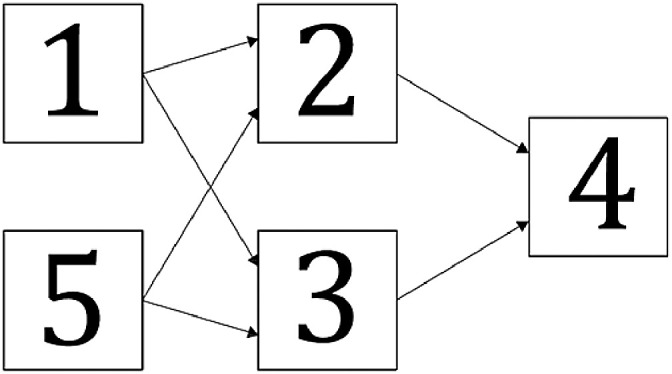
System scheme example.
• Redundant: the user must specify here which component is a redundant of the described one. The field must be filled with “0” in case of no redundancies, or with the components number, also separated by underline, in case of more than one redundancy. In Fig. 1, component 5 is a redundancy of component 1. In this case, for component 1, this field should be filled with “5”;
• AffectedBy: the components informed here are those that affect the component that the user is giving information about. For instance, in Fig. 1, component 4 is affected by components 2 and 3, and therefore this field would be filled with “2_3” for component 4. Component 1 would have this field filled by “0”;
• Function: focusing on cogeneration and power plants, the user must inform here one of the allowed values: “electricity”, “thermal”, or “none”;
• AverageRepairTime: the repair time is treated as a normal distribution in the simulation. The content of this field represents the average of this distribution for the described component, with 20% of standard deviation. The purpose of considering this behavior is to include the uncertainty of the failure origin.

The first step to be followed by the user is to describe the system(s) that will be analyzed. This procedure is done in “S#()_info.xlsx” file, in the cells highlighted in green. General information is given in the green cells on the right: system identification number, number of components, number of components generating electricity and number of components generating thermal energy. The components specific characterization takes into account the following fields that need to be filled in:

• Type: it is basically the name or the abbreviation to identify each component. It does not interfere in the simulation, being only an identification tag;
• Affects: in this field, the user has to inform the components (by their numbers, not types) that are affected by the component that the user is giving information about, or fill with “0” in case it does not affect another. The numbers need to be separated by underline. As example in Fig. 1, the component 1 affects components 2 and 3. Therefore, for component 1, the field “affects” must be filled with “2_3”;

Once the description is finished, the user must name this file as “S#{IdentificationNumber}_info.xlsx” and save it in the folder that contains the Python program. This procedure can be done as many times as the user wants before simulation, depending on the number of systems that will be analyzed.

### 2.2 Simulation

Once all the systems are described and saved in their respective archives, the user must open the “resilience analysis.py” file in a Python Integrated Development Environment, install the *xlrd* and *xlwt* packages and run the program. It is important that the Python program and the file describing the system(s) are in the same folder. The inputs are:

• System(s) ID: identification number(s) of the system(s) that the user wants to simulate. In case of more than one system, the numbers must be written separated by comma;
• Lifetime (*T*): expected system(s) lifetime, in hours;
• Number of simulations (*N*): number of times that the program will simulate a certain lifetime. According to [1], 3000 simulations are sufficient to stabilize the coefficient of variation in this method;
• Probability of component normal operation (*p_i_*): the complementary of failure probability. This value is assumed for all components;
• Component unsuccessful repair probability (p_cnr_): the complementary of repair probability. This value is also assumed for all components.

The simulation will start as soon as the user informs the parameters above. At the end, the program will generate a .xls file with simulated data for each given system. This data includes the simulation time, which is the total counted time, including during plant shutdown; resilient time, the time in which the system maintained its operation with failed components; and downtime, representing the plant shutdown period recovering from a failed situation with no energy generation. This file will be named according to the system ID and number of simulations defined by the user.

Each step in the code is commented, so that the user is able to understand in detail the functionality of the simulation. In addition to clarify the procedure, the comments also have the purpose of providing the user possibilities of changing the code according to the objective of her/his own investigation.

### 2.3 Data processing

**Fig. 2:**
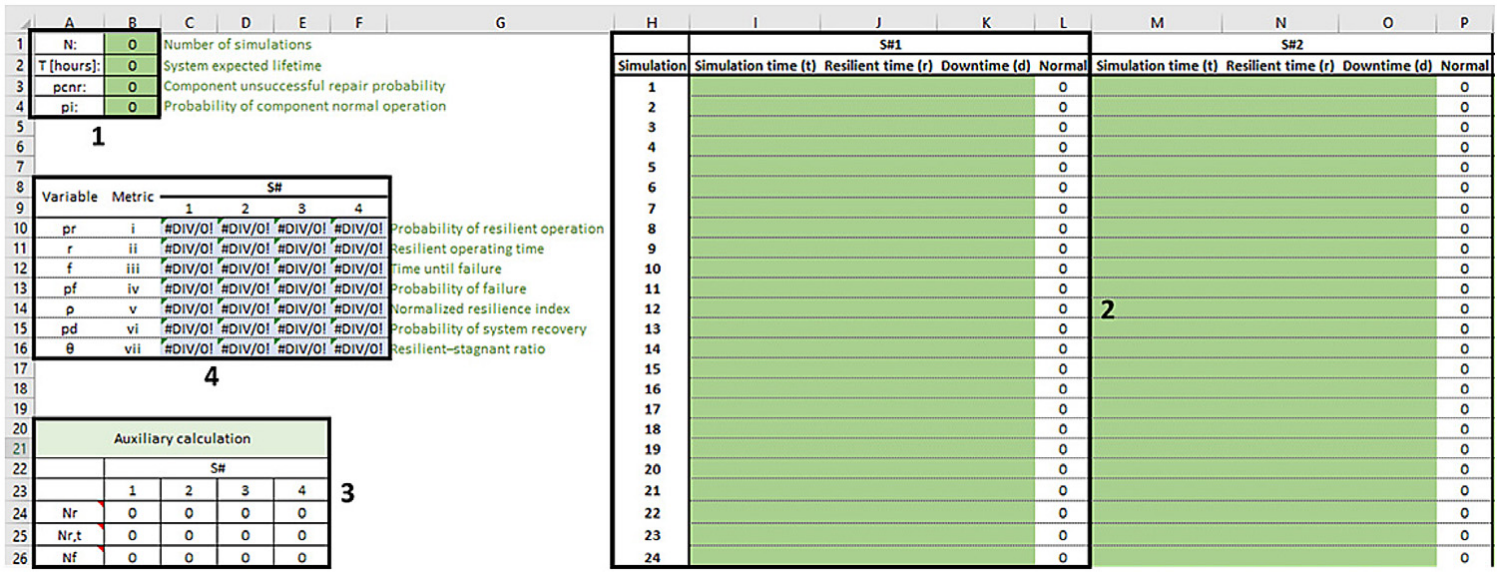
Illustration of the “Metrics calculation.xlsx” file.

With the simulation completed, the “Metrics calculation.xlsx” file must receive the data from the archive originated by the simulation. Fig. 2 exhibits the “Metrics calculation.xlsx” file with four marks. In the first one, the parameters used in the simulations are required for the resilience evaluation. The data generated by the simulation is placed in the green cells within mark 2, which is adjusted to receive 3000 simulations, although the user is able to increase or decrease this value according to his/her aim. There are four spaces to receive data from four different systems, which is also changeable. As soon as this data is given to the file, the cells within mark 3 calculates some auxiliary variables, which support the metrics placed in mark 4. In this file, it is advisable not to change information of the cells in marks 3 and 4, which are responsible for calculating the metrics for resilience analysis.

## CRediT Author Statement

**Fellipe Sartori da Silva:** Methodology, Software, Validation, Formal analysis, Investigation, Resources, Data Curation, Writing - Original Draft, Writing - Review & Editing, Visualization, Funding acquisition; **José Alexandre Matelli:** Conceptualization, Methodology, Software, Validation, Formal analysis, Resources, Writing - Review & Editing, Supervision, Project administration, Funding acquisition.

## Declaration of Competing Interest

The authors declare that they have no known competing financial interests or personal relationships which have, or could be perceived to have, influenced the work reported in this article.
