# Corrigendum to ‘Script for resilience analysis in energy systems: Python programming code and partial associated data of four cogeneration plants’ [Data in Brief 36 (2021) 106986]

**DOI:** 10.1016/j.dib.2021.107689

**Published:** 2021-12-07

**Authors:** Fellipe Sartori da Silva, José Alexandre Matelli

**Affiliations:** São Paulo State University, School of Engineering, Department of Chemistry and Energy, Guaratinguetá, São Paulo, Brazil

The authors would like to inform that a mistake in the simulation codes was found. As soon as it was detected, it was readily corrected. The authors regret that the simulation codes with this error are published by the referred paper [1], and would like to replace them by the revised ones. The purpose is to maintain the good scientific practice and spread the appropriate authentic method.

The error was related to failure and repair propagation processes. Once a component failed, the program was not able to correctly propagate the fail to other components, which altered the results of the related paper – for which we also sent a corrigendum.

The corrected codes and the new data are attached to the new version of the file, found in Mendeley Repository [2].

The authors would like to apologise for any inconvenience caused.
